# Cerebral Blood Volume ASPECTS Is the Best Predictor of Clinical Outcome in Acute Ischemic Stroke: A Retrospective, Combined Semi-Quantitative and Quantitative Assessment

**DOI:** 10.1371/journal.pone.0147910

**Published:** 2016-01-29

**Authors:** Marina Padroni, Andrea Bernardoni, Carmine Tamborino, Gloria Roversi, Massimo Borrelli, Andrea Saletti, Alessandro De Vito, Cristiano Azzini, Luca Borgatti, Onofrio Marcello, Christopher d’Esterre, Stefano Ceruti, Ilaria Casetta, Ting-Yim Lee, Enrico Fainardi

**Affiliations:** 1 Section of Neurology, Department of Biological, Psychiatric and Psychological Science, University of Ferrara, Ferrara, Italy; 2 Section of Diagnostic Imaging, Department of Morphology, Surgery and Experimental Medicine, University of Ferrara, Ferrara, Italy; 3 Neuroradiology Unit, Department of Neuroscience and Rehabilitation, Azienda Ospedaliera Universitaria, Ferrara, Italy; 4 Neurology Unit, Department of Neuroscience and Rehabilitation, Azienda Ospedaliera Universitaria, Ferrara, Italy; 5 Calgary Stroke Program, Department of Clinical Neurosciences, University of Calgary, Calgary, Alberta, Canada; 6 Imaging Research Lab, Robarts Research Institute, Western Ontario University, London, Ontario, Canada; 7 Imaging program, Lawson Health Research Institute, Western Ontario University, London, Ontario, Canada; National Cheng Kung University, TAIWAN

## Abstract

**Introduction:**

The capability of CT perfusion (CTP) Alberta Stroke Program Early CT Score (ASPECTS) to predict outcome and identify ischemia severity in acute ischemic stroke (AIS) patients is still questioned.

**Methods:**

62 patients with AIS were imaged within 8 hours of symptom onset by non-contrast CT, CT angiography and CTP scans at admission and 24 hours. CTP ASPECTS was calculated on the affected hemisphere using cerebral blood flow (CBF), cerebral blood volume (CBV) and mean transit time (MTT) maps by subtracting 1 point for any abnormalities visually detected or measured within multiple cortical circular regions of interest according to previously established thresholds. MTT-CBV ASPECTS was considered as CTP ASPECTS mismatch. Hemorrhagic transformation (HT), recanalization status and reperfusion grade at 24 hours, final infarct volume at 7 days and modified Rankin scale (mRS) at 3 months after onset were recorded.

**Results:**

Semi-quantitative and quantitative CTP ASPECTS were highly correlated (p<0.00001). CBF, CBV and MTT ASPECTS were higher in patients with no HT and mRS≤2 and inversely associated with final infarct volume and mRS (p values: from p<0.05 to p<0.00001). CTP ASPECTS mismatch was slightly associated with radiological and clinical outcomes (p values: from p<0.05 to p<0.02) only if evaluated quantitatively. A CBV ASPECTS of 9 was the optimal semi-quantitative value for predicting outcome.

**Conclusions:**

Our findings suggest that visual inspection of CTP ASPECTS recognizes infarct and ischemic absolute values. Semi-quantitative CBV ASPECTS, but not CTP ASPECTS mismatch, represents a strong prognostic indicator, implying that core extent is the main determinant of outcome, irrespective of penumbra size.

## Introduction

Acute ischemic stroke (AIS) can be effectively treated with reperfusion therapies, such as intravenous fibrinolysis with recombinant tissue-plasminogen activator (t-PA) and mechanical thrombectomy, which improve clinical outcome [1]. Moreover, the selection of patients with AIS for reperfusion therapies is presently suboptimal as many patients do not achieve a good clinical outcome, despite successful recanalization [2]. This is largely due to the constraints of neuroimaging techniques which are unable to accurately identify patients who could benefit from such therapies [3]. In fact, non-contrast CT (NCCT) using the Alberta Stroke Program Early CT Score (ASPECTS) to detect early ischemic signs in the middle cerebral artery (MCA) territory is still considered the method of choice for the selection of patients eligible for reperfusion therapies [4]. However, while ASPECTS prognostic value has been questioned, ASPECTS does not yield the extent of ischemic penumbra and its inter-observer agreement is modest [5].

Using a threshold-based paradigm, CT perfusion (CTP) has the potential to discriminate between irreversibly damaged tissue, the infarct core (the area with low CBV and low CBF but high MTT), and tissue at risk of infarction, the ischemic penumbra (the area with normal CBV and low CBF but high MTT) [6,7]. Given that AIS patients receiving treatment according to this penumbral hypothesis were shown to have a good prognosis, it suggests a possible role for CTP in the selection of patients for reperfusion therapies [3]. Unfortunately, these results were not confirmed by previous clinical trials based on penumbral selection in that a favorable CTP penumbra profile was not significantly associated with a better outcome [8,9]. The demonstration that CBF better approximates the extent of the infarct core than CBV could be the explanation for these negative findings [10]. In addition, this new approach was successfully used for patient selection for endovascular treatment in two recent clinical trials [11,12]. In this context, the application of ASPECTS methodology to CTP maps could be useful to overcome the limitations of threshold-derived CTP penumbral maps based on MTT/CBV mismatch model because it has been proven that CTP ASPECTS has a strong correlation with outcome [13–17], a prognostic value greater than NCCT ASPECTS [13–15] and a good interobserver agreement [18]. Furthermore, although previous publications indicate that CBV ASPECTS is the best predictor of prognosis, the optimal threshold to discriminate between AIS patients with good and poor clinical outcome ranges from 6 to 9 [13–17]. Finally, the accuracy of visual CTP ASPECTS abnormalities in properly identifying the severity of ischemia remain to be understood. In particular, it is still unclear whether CBV and CBF or MTT ASPECTS defects correspond to their respective absolute and relative values which are currently presumed to be indicative of infarct core and total hypoperfusion, respectively [6,7]. In light of these considerations, the objective of this retrospective study was two-fold: to clarify the prognostic relevance of CTP ASPECTS and to determine whether CTP ASPECTS is able to recognize the severity of hypoperfusion, as compared with quantitative measurements.

## Materials and Methods

### Ethics statement

The study was approved by the Research Ethics Board of Azienda Ospedaliero-Universitaria di Ferrara (Italy). Written informed consent was obtained from each patient or from the patient’s legal representative at admission.

### Patient selection and study design

**Fig 1:**
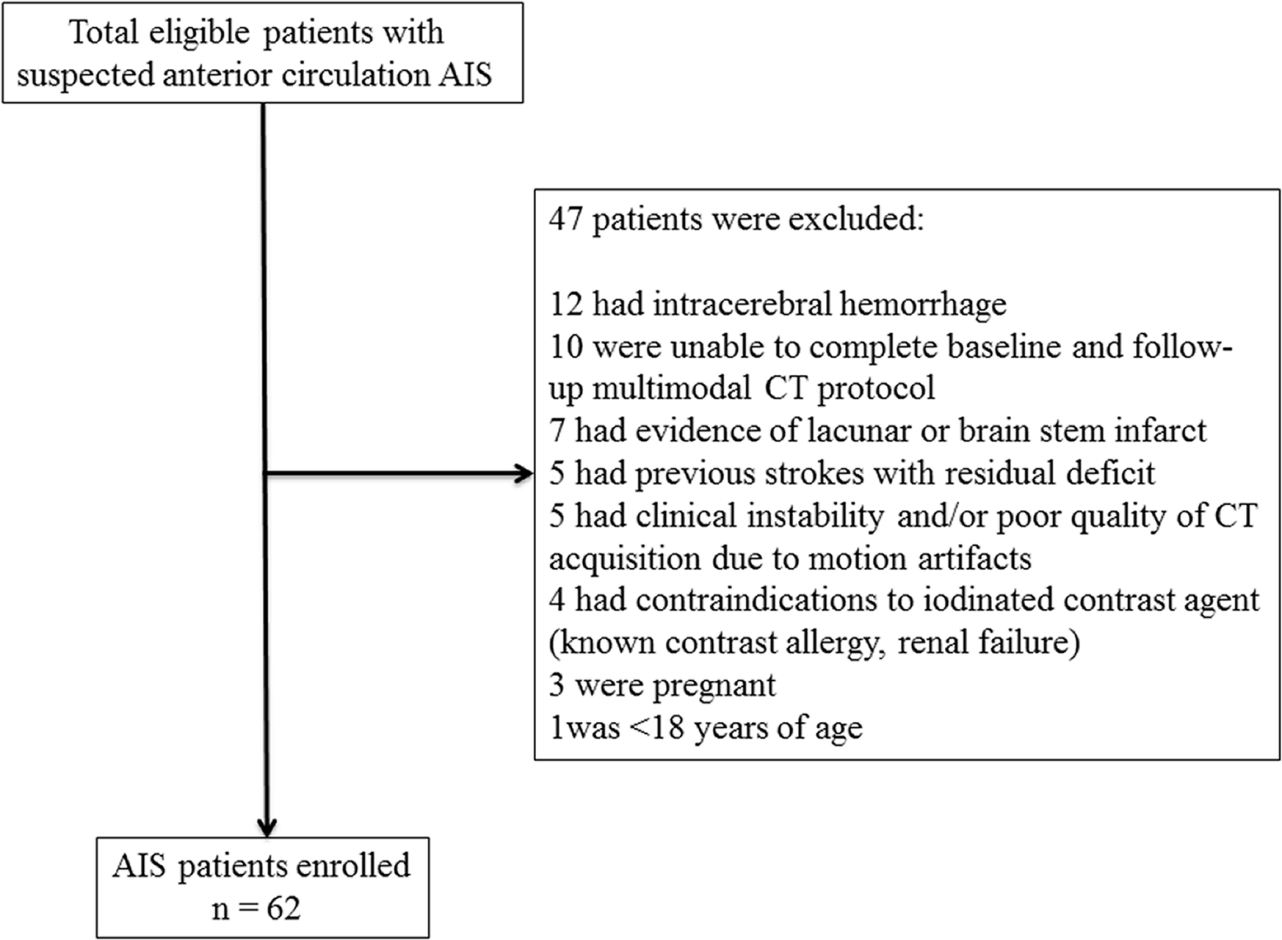
Inclusion and exclusion flow chart of the study.

At our Institution, all patients presenting with suspected anterior circulation AIS and no history of renal failure or contrast allergy routinely undergo NCCT, CT angiography (CTA) of the cervical and intracranial vessels and CTP at admission if they arrive at the hospital within 8 hours of symptom onset. If diagnosis of AIS is confirmed by neuroimaging findings, NCCT, CTA and CTP are repeated at 24 hours to determine initial infarct size, degree of recanalization and extent of reperfusion, respectively, and an additional NCCT is performed at 7 days to determine the final infarct volume. If appropriate, according to the current recommended guidelines [1], AIS patients receive intravenous fibrinolysis with t-PA or endovascular treatment when stroke onset is <4.5 hours or between 4.5–8 hours, respectively. We retrospectively reviewed the clinical and radiological data of 109 consecutively screened potentially eligible AIS patients admitted to the Neuroscience Department of Ferrara during the period from October 2008 to October 2010. This time period was chosen in order to analyze only patients with one-phase CTP acquisition protocol, thus allowing a comparison with previous studies [13–17]. Inclusion criteria were: 1) presentation at the hospital within 8 hours from symptom onset; 2) baseline and follow-up confirmation of hemispheric AIS; 3) baseline and follow-up CT imaging carried out at the established time-points after symptom onset (NCCT, CTA and CTP at admission, NCCT, CTA and CTP at 24 hours and NCCT at 7 days); 4) CTP performed with a one-phase acquisition protocol. Exclusion criteria were: 1) detection of intracerebral hemorrhage at admission NCCT; 2) inability to complete multimodal CT protocol at baseline and follow-up; 3) evidence of lacunar or brain stem infarct; 4) previous strokes with residual deficit; 5) contraindications to iodinated contrast agent; 6) pregnancy; 7) age <18 years; 8) clinical instability and/or poor quality of CT acquisition due to motion artifacts. Overall, 62 patients with clinical diagnosis of AIS met the previously mentioned inclusion criteria and were included for the study (Fig 1).

### Clinical assessment

Type of reperfusion therapy (if any) was also collected. Stroke subtypes were classified according to Trial of ORG 10172 in Acute Stroke Treatment (TOAST) criteria. Disease severity at onset, at 24 hours and at 7 days at 3 months after stroke was scored using the National Institutes of Health Stroke Scale (NIHSS). Clinical outcome was measured by the modified Rankin scale (mRS) at 3 months. mRS≤2 and >2 were defined as good and poor outcomes, respectively.

### Imaging acquisition protocol

All imaging was conducted on a 64-slice Lightspeed VCT scanner (GE Healthcare, Waukesha, WI, USA). NCCT helical scans were performed from the skull base to the vertex using the following imaging parameters: 120 kV, 340 mA, 4x5-mm collimation, 1 second/rotation, and table speed of 15 mm/rotation. CTA was performed as follows: 0.7 mL/kg contrast (maximum 90 mL), 5- to 10-second delay from injection to scanning, 120 kV, 270 mA, 1 second/rotation, 1.25-mm thick slices, and table speed 3.75 mm/rotation. CTA covered from the carotid bifurcation to vertex. CTP studies were performed at admission with a dynamic first-pass bolus-tracking methodology according to a one-phase imaging protocol consisting of an acquisition of 50-seconds continuous (cine) scans reconstructed with a 25 cm field-of-view at 0.5 second intervals to produce a series of 99 sequential images for each of eight 5 mm thick sections (792 images; matrix size = 512 × 512) which covered a total of 4 cm from the basal ganglia to the lateral ventricles. The other parameters of the cine scan were 80 kV, 100 mA and 0.5 rotation time. The cine scan was initiated 5 seconds after the automatic injection (Medrad, Indianola, PA) of 40 ml of non-ionic contrast agent (Iomeron 300 mg/ml, Bracco Imaging SpA) at the rate of 4 ml/sec into an antecubital vein.

### Imaging processing and analysis

**Fig 2:**
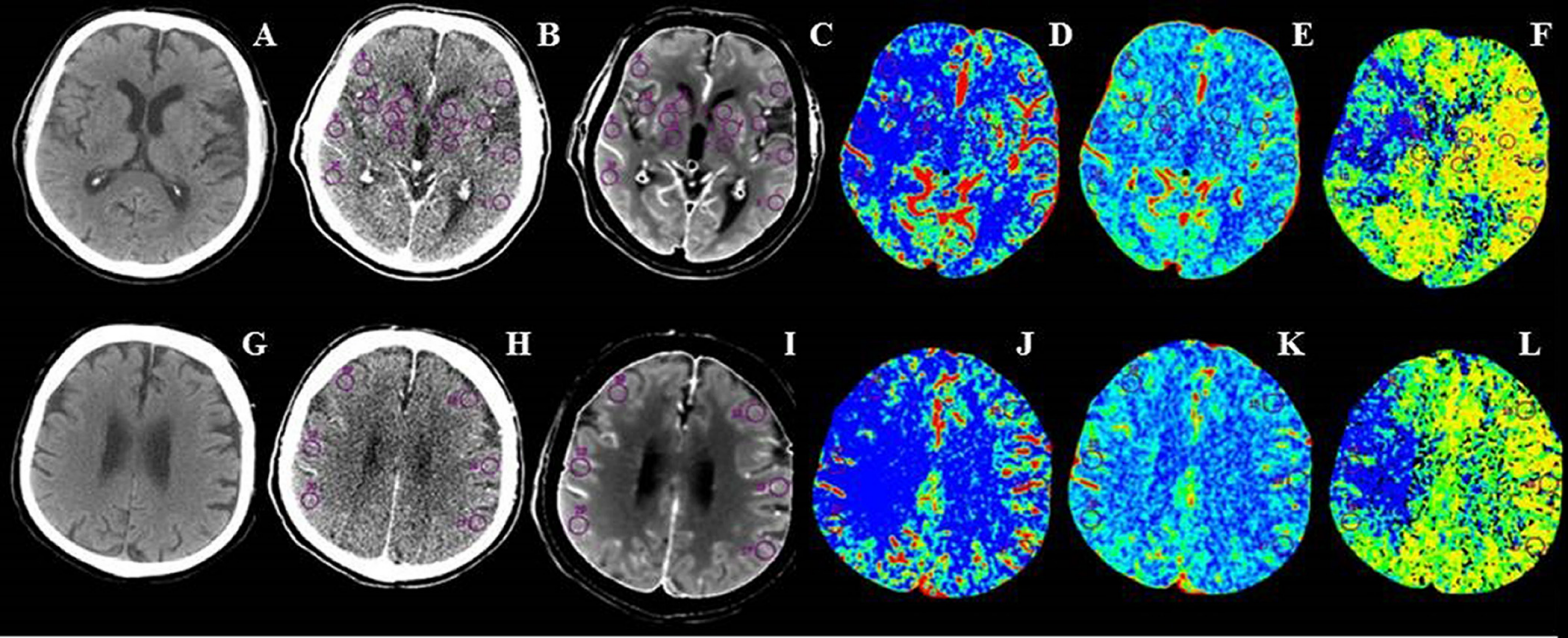
Non-constrast CT, baseline source and average CT perfusion images, cerebral blood flow (CBF), cerebral blood volume (CBV) and mean transit time (MTT) maps at ganglionic (A-F) and supraganglionic (G-L) axial ASPECTS levels. Multiple circular regions of interest (ROIs) larger than 1 cm^2^ placed freehand in the affected hemisphere and automatically reflected into homologous regions of the contralateral hemisphere were used to measure CBF, CBV and MTT absolute values from the corresponding functional maps in ASPECTS regions at ganglionic (anterior middle cerebral artery cortex, middle cerebral artery cortex lateral to insular ribbon, posterior middle cerebral artery cortex, insula, caudate nucleus, lentiform nucleus and internal capsule) and supraganglionic (anterior, lateral, and posterior middle cerebral artery cortical territories immediately superior to the previous ones, rostral to basal ganglia) sections.

The extension of early ischemic changes (hypoattenuation, loss of the gray-white matter boundary and effacement of cortical sulci) was evaluated on NCCT at onset by ASPECTS methodology, a 10-point ordinal scale that rates the presence or absence of ischemia in 10 regions included in the middle cerebral artery territory assigning a score of 1 for normal and 0 for a region showing early ischemic signs [4]. According to EXTEND-IA trial [11], the severity of arterial occlusion was judged on CTA at onset using a modified version of the Thrombolysis in Myocardial Infarction (TIMI) grading system [19]: complete occlusion (TIMI score = 0–1); partial occlusion (TIMI score = 2); no occlusion (TIMI score = 3). Patients were categorized as occluded if TIMI score was between 0 and 1 or not occluded when TIMI score was between 2 and 3. In occluded patients, the site of occlusion was assessed as indicated before [20]. All CTP scans were assessed using a commercially available delay-sensitive deconvolution software (CT Perfusion 3, GE Healthcare, Waukesha, WI). For each CTP scan, time-density curves (TDC) for the arterial input function (AIF) and venous output functions (VOF) were obtained from the anterior cerebral artery and superior sagittal sinus, respectively. The AIF was corrected for partial volume averaging using the VOF-TDC. CBF, CBV and MTT CTP maps were generated for each patient by deconvolution of tissue TDCs and the AIF. CBF, CBV and MTT values were expressed in ml·min^-1^·(100g)^-1^, ml·(100g)^-1^and seconds, respectively. Average CTP maps were created by averaging the cine CTP images over the duration of the first pass of contrast. These average CTP images were used to exclude cerebrospinal fluid and cranium from analysis. Large blood vessels were automatically excluded from calculation by the software. Color coded functional CTP map scales were set at 0–100 ml·min^-1^·(100g)^-1^ for CBF, 0–8 ml·(100g)^-1^ for CBV and 0–20 seconds for MTT. The side of the hypoperfused arterial territory, as visually detected on MTT maps at admission, was indicated for each patient. ASPECTS was semi-quantitatively and quantitatively calculated using CBF, CBV and MTT CTP maps. As described elsewhere [13–17], semi-quantitative analysis was performed by applying ASPECTS and allotting a score of 1 for normal and 0 for abnormal perfusion regions. Specifically, we subtracted 1 point for each affected area, specified by ASPECTS methodology [4], where CBF, CBV or MTT defects were visually detected. As shown in Fig 2, quantitative assessment was carried out by measuring CTP values within multiple circular regions of interest (ROIs) larger than 1 cm^2^ and manually drawn on the baseline source and averaged CTP images on all specified regions of the two ASPECTS levels in the ischemic hemisphere. ROI’s were then automatically reflected about the midline to obtain homologous ROIs in the contralateral hemisphere. All these ROIs were automatically superimposed on CBF, CBV and MTT colour maps. One point was subtracted for each ROI with abnormal CBF, CBV and MTT levels according to the previously established thresholds: CBF≤24.6 ml·min^-1^·(100g)^-1^; CBV≤1.1 ml·(100g)^-1^; MTT>145% of contralateral side [6,7]. As MTT and CBV maps were used to define total hypoperfusion and infarct core, respectively, CTP ASPECTS mismatch was considered as the CBV ASPECTS minus the MTT ASPECTS in both semi-quantative and quantitative analyses [21]. Final infarct volume was measured on follow-up NCCT at 7 days after symptom onset with a multi-slice planimetric method by summation of the hypodense areas, manually traced on each slice in which they were detectable, multiplied by slice thickness. As previously described [22], swelling extending across midline or producing ventricular effacement were excluded from calculation to reduce early subacute vasogenic edema effects. The type of HT was classified on NCCT at 24 hours and at 7 days post ictus according to the European Cooperative Acute Stroke Study (ECASS)-II criteria [23] into four different categories: hemorrhagic infarction type 1 (HI-1), HI type 2 (HI-2), Parenchymal hemorrhage type 1 (PH-1), and PH type 2 (PH-2). In agreement with EXTEND-IA trial [11], recanalization was scored on CTA at 24 hours with an adaptation of the TIMI grading system [19]: persistent occlusion (TIMI score = 0–1); partial recanalization (TIMI score = 2); full recanalization (TIMI score = 3). Patients with TIMI score ranging from 2 to 3 were considered as recanalized, whereas patients with TIMI score of 0 and 1 were classified as not recanalized. All patients not occluded on CTA at admission had a TIMI score of 3 at 24 hour-CTA and therefore, were considered as recanalized. As only 9 patients underwent endovascular procedures, recanalization status was established using CTA at 24 hours also in these subjects to make data collection from our study population more homogeneous. Reperfusion was evaluated by reperfusion index that measures the percentage reduction of baseline MTT lesion at 24 hours [24]. For this purpose, visually identified MTT defect volume was obtained by a manual multi-slice planimetric method at admission and at 24 hours. Patients with a reperfusion index >75% were considered as reperfused.

### Statistical analysis

The normality of each variable was checked by using the Kolmogorov-Smirnov test and parametric or non- parametric tests were used accordingly. Continuous variables were compared by independent-sample t test or Mann-Whitney U test, whereas their correlations were assessed by linear regression or Spearman's rank correlation coefficient, respectively. Receiver Operating Characteristic (ROC) curves were used to determine semi-quantitative and quantitative CTP ASPECTS cut-off points which optimally discriminated between good and poor outcome. A value of p<0.05 was accepted as statistically significant.

## Results

### Clinical and radiological findings

Demographic, clinical and radiological data recorded in our AIS patients are listed in Table 1. In our study population, 37/62 (59.7%) patients were treated (28 with intravenous thrombolysis with r-tPA, 3 with intra-arterial thrombolysis and 6 with mechanical thrombectomy), whereas 25/62 (40.3%) did not receive any therapy due to the presence of hypodensity >1/3 cerebral hemisphere, as indicated by ASPECTS≤7, on admission NCCT (n = 19), the occurrence of severe stroke, as showed by a NIHSS>25 (n = 4) at presentation and the current use of anticoagulant with INR>1.7 (n = 2). A good outcome (mRS≤2) was achieved in 42/62 (67.7%) cases, whereas a poor outcome (mRS>2) was present in 20/62 (32.3%) patients. HT was observed in 27/62 (43.5%) patients (22 HI and 5 PH) appearing at 24 hours (n = 8) and at 7 days (n = 19) after stroke onset. At twenty-four hour post ictus, 45/62 (72.6%) achieved recanalization while 17/62 (27.4%) did not, 25/62 (40.3%) were reperfused and 37/62 (59.7%) not reperfused.

**Table 1:** Demographic, clinical and radiological characteristics in 62 patients with acute ischemic stroke.

Sex: Female/Male	32/30
Age, years: mean±SD	68.1±11.6
TOAST:	
*Atherotrombotic*	30/62 (48.4%)
*Cardioembolic*	28/62 (45.2%)
*Undetermined*	4/62 (6.4%)
NIHSS at entry: median, IQR, mean±SD, range	11, 8–17, 12.5±6.9, 1–28
NIHSS at 24 hours: median, IQR, mean±SD, range	8, 3–13, 9.1±6.9, 0–26
NIHSS at 7 days: median, IQR, mean±SD, range	4, 1–11, 6.9±6.9, 0–25
mRS at 3 months: median, IQR, mean±SD, range	1, 1–3, 2.0±1.6, 0–6
Time between symptom onset and CT scan at admission (hours): median, IQR, mean ± SD, range	2, 1.3–4.0, 2.8±2.1, 0.4–7.5
Time window at admission: n/total (%)	
*< 4*.*5 hours*	48/62 (77.4%)
*4*.*5–8 hours*	14/62 (22.6%)
NCCT ASPECTS at admission: median, IQR, mean±SD, range	9, 7–10, 8.5±1.6, 4–10
Occlusion grade on CTA at admission: n/total (%)	
*Occluded*	45/62 (72.6%)
*Not occluded*	17/62 (27.4%)
Occlusion location on CTA at admission: n/total (%)	
*Proximal MCA-M1*	7/62 (11.3%)
*Distal MCA-M1*	12/62 (19.4%)
*MCA-M2*	19/62 (30.6%)
*Proximal ICA*	4/62 (6.5%)
*T occlusion*	3/62 (4.8%)
Involved arterial territory on CTP at admission: n/total (%)	
*Right MCA*	39/62 (62.9%)
*Left MCA*	23/62 (37.1%)
Time between symptom onset and CT scan at 24 hours (hours): median, IQR, mean±SD, range	23, 20–24, 21.6**±** 3.1, 12–24
Time between CT scan at admission and at 24 hours (hours): median, IQR, mean±SD, range	24, 22–24, 22.0±2.0, 17–24
Time between symptom onset and CT scan at 7 days (days): median, IQR, mean±SD, range	6, 6–7, 6.3±0.9, 5–9
Final infarct volume (ml) on NCCT at 7 day: median, IQR, mean±SD, range	24, 7–54, 44.5±56,2, 0–245

SD = Standard deviation; TOAST = Trial of ORG 10172 in Acute Stroke Treatment; NIHSS = National Institutes of Health Stroke Scale; IQR = Interquartile range; mRS = modified Rankin Scale: NCCT = non-contrast CT; CTA = CT angiography; MCA = middle cerebral artery; ICA = Internal carotid artery: CTP = CT perfusion.

### Relationship between semi-quantitative and quantitative ASPECTS

**Fig 3:**
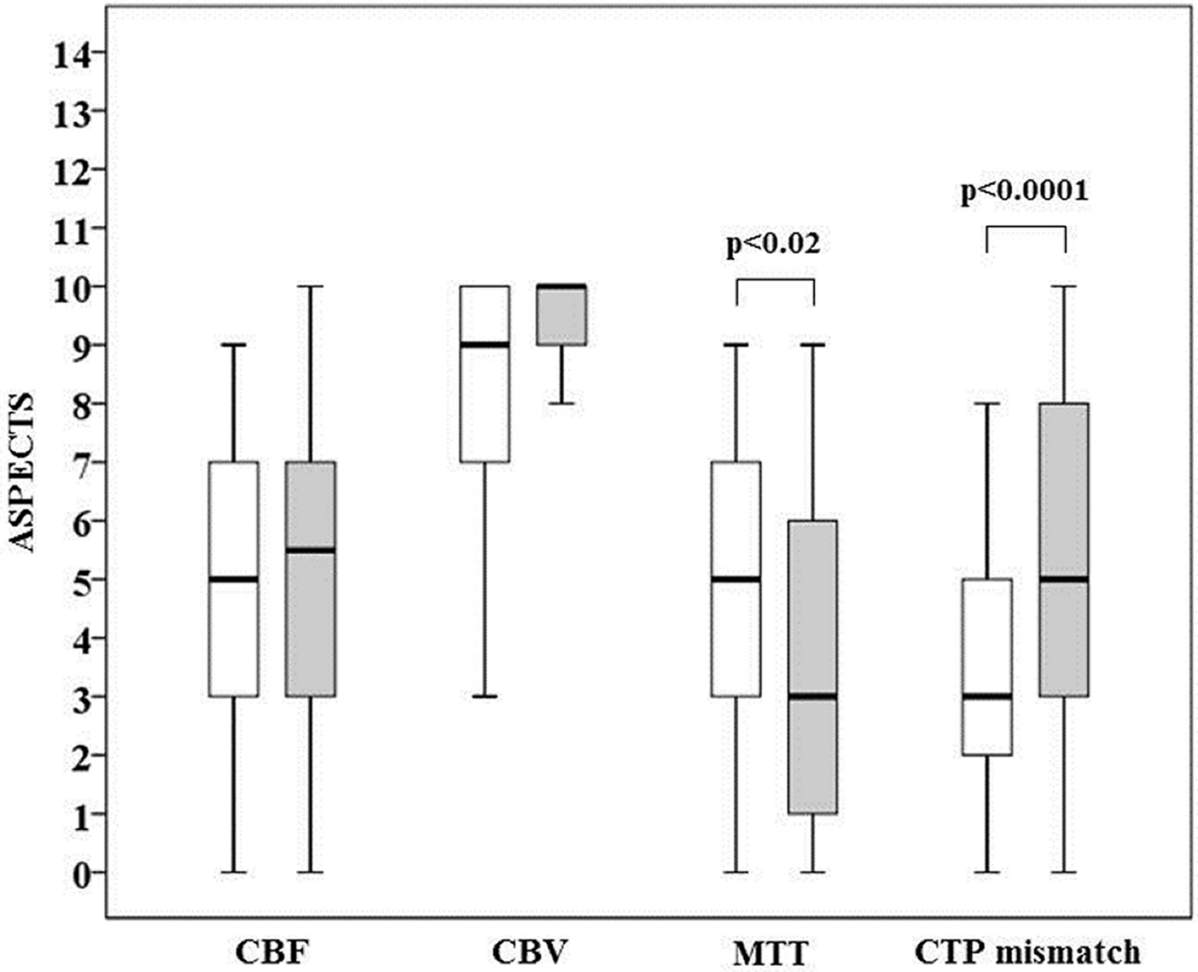
Comparison between semi-quantitative (white boxes) and quantitative (grey boxes) ASPECTS for cerebral blood flow (CBF), cerebral blood volume (CBV), mean transit time (MTT) and CT perfusion (CTP) mismatch. The boundaries of the box represent the 25th-75th quartile. The line within the box indicates the median. The whiskers above and below the box correspond to the highest and lowest values, excluding outliers.

**Fig 4:**
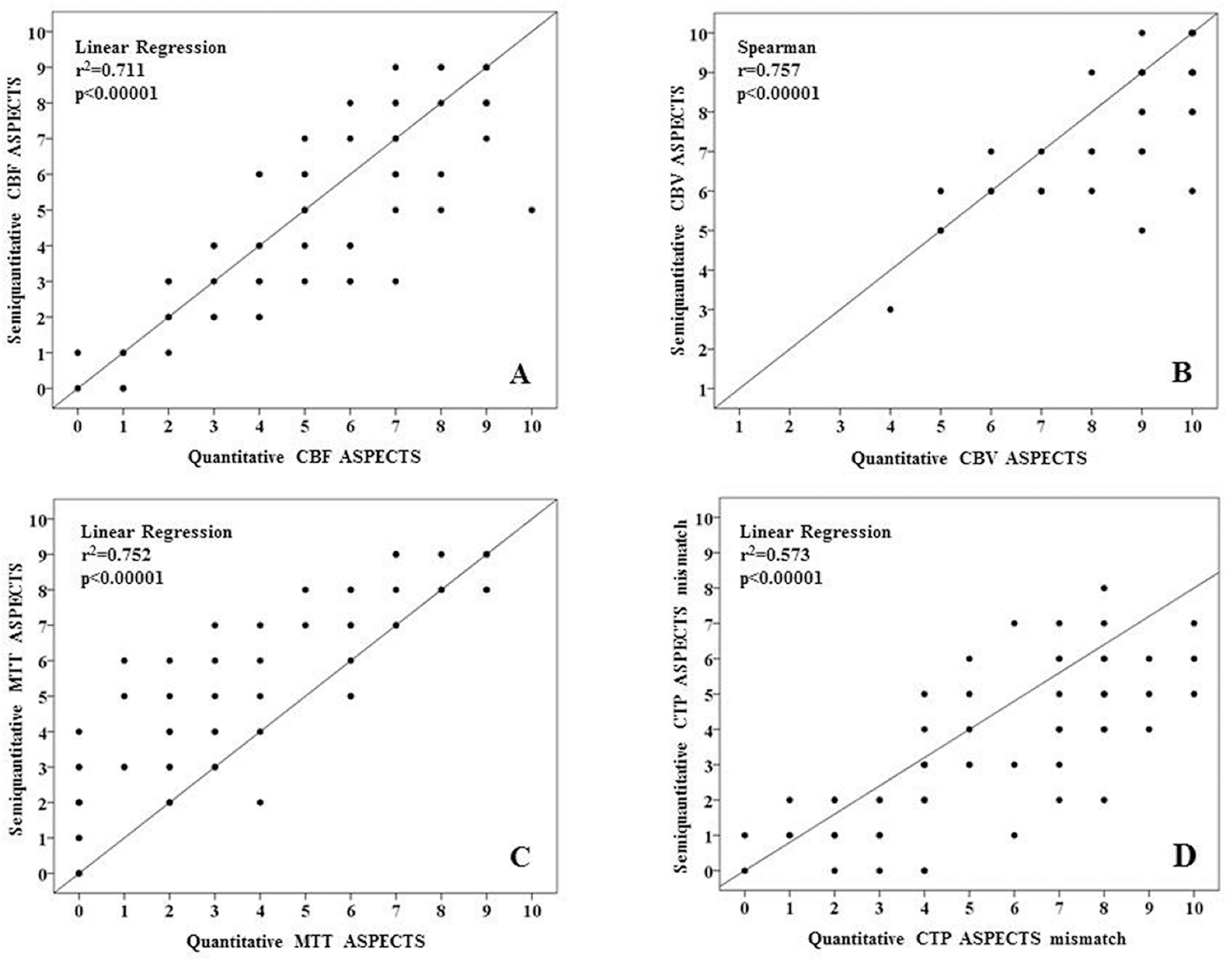
Relationships between semi-quantitative (y-axis) and quantitative (x-axis) ASPECTS for cerebral blood flow (CBF) (*panel A*), cerebral blood volume (CBV) (*panel B*), mean transit time (MTT) (*panel C*) and CT perfusion (CTP) mismatch (*panel D*).

Median semi-quantitative ASPECTS was 5 (IQR = 3–7; mean±SD = 4.9±2.7; range = 0–9) for CBF maps, 9 (IQR = 7–10; mean±SD = 8.3±2.7; range = 3–10) for CBV and 5 (IQR = 3–7; mean±SD = 5.0±2.7; range = 0–9) for MTT. Median semi-quantitative ASPECTS mismatch was 3 (IQR = 2–5; mean±SD = 3.3±2.1; range = 0–8). Median quantitative ASPECTS was 5.5 (IQR = 3.3–7; mean±SD = 5.0±3.0; range = 0–10) for CBF maps, 10 (IQR 9–10; mean±SD = 8.9±1.6; range = 4–10) for CBV and 3 (IQR = 1.3–6; mean±SD = 3.7±2.9; range = 0–9) for MTT. Median quantitative ASPECTS mismatch was 5 (IQR = 3–8; mean±SD = 5.2±2.7; range = 0–10). There were no differences between semi-quantitative and quantitative CBF and CBV ASPECTS, whereas ASPECTS was greater for semi-quantitative than for quantitative MTT (p<0.02) and for quantitative than for semi-quantitative mismatch (p<0.0001) (Fig 3). As illustrated in Fig 4, semi-quantitative and quantitative CBF, CBV, MTT ASPECTS and CTP ASPECTS mismatch were highly correlated (p<0.00001).

### Relationship between semi-quantitative and quantitative ASPECTS and clinical and radiological features

As reported in Table 2, AIS patients with no HT had higher values compared with those with HT for semi-quantitative and quantitative CBF (p<0.0001), CBV (p<0.00001 and p<0.001, respectively) and MTT (p<0.001 and p<0.0001, respectively) ASPECTS. On the contrary, while semi-quantitative CTP ASPECTS mismatch was similar in AIS patients with and without HT, quantitative CTP ASPECTS mismatch was more elevated in AIS patients with no HT than in those with HT (p<0.05). Semi-quantitative and quantitative ASPECTS were greater in AIS patients with good outcome than in those with poor outcome for CBF (p<0.01 and p<0.001, respectively), CBV (p<0.01 and p<0.05, respectively) and MTT (p<0.02 and p<0.001, respectively). No differences were found between AIS patients with mRS≤2 and mRS>2 for semi-quantitative CTP ASPECTS mismatch, whereas AIS patients with good outcome had more elevated quantitative CTP ASPECTS mismatch compared to those with poor outcome (p<0.02). Tables 3 and 4 show the correlations we found between semi-quantitative and quantitative CTP ASPECTS and radiological and clinical outcome measures respectively. An inverse association was observed between semi-quantitative and quantitative CBF, CBV and MTT ASPECTS and final infarct volume (p<0.00001) as well as between mRS and semi-quantitative and quantitative ASPECTS for CBF (p<0.01 and p<0.0001, respectively), CBV (p<0.001 and p<0.0001, respectively) and MTT (p<0.02 and p<0.0001, respectively). No relationships were detected between semi-quantitative CTP ASPECTS mismatch and final infarct volume and mRS, whereas quantitative CTP ASPECTS was positively but only modestly correlated with both final infarct volume and clinical outcome (p<0.02 and 0.05, respectively). After categorization of AIS patients according to recanalization status, a negative association was observed between final infarct volume and semi-quantitative and quantitative CBF (p<0.001 and p< 0.01, respectively), CBV (p<0.00001) and MTT (p<0.01 and p<0.001, respectively) ASPECTS, and between clinical outcome and qualitative MTT ASPECTS and CTP mismatch (p<0.01) in recanalized patients. No further relationships were seen between CTP ASPECTS and final infarct volume and clinical outcome in recanalized and not recanalized patients. When AIS patients were stratified according to reperfusion status, an inverse correlation was found between final infarct volume and semi-quantitative CBF (p<0.01), semi-quantitative and quantitative CBV (p<0.02 and p<0.01, respectively) and MTT (p<0.02) ASPECTS in reperfused patients and between final infarct volume and semi-quantitative and quantitative CBF (p<0.01), CBV (p<0.000001 and p<0.0001, respectively) and MTT (p<0.05 and p<0.01, respectively) ASPECTS in not reperfused patients. mRS was negatively associated with semi-quantitative and quantitative CBF (p<0.02 and p<0.01, respectively) and CBV (p<0.01) and quantitative MTT (p<0.02) ASPECTS only in not reperfused patients. There were no additional relationships between CTP ASPECTS and final infarct volume and clinical outcome in reperfused and not reperfused patients.

**Table 2:** Semi-quantitative and quantitative CT perfusion (CTP) Alberta Stroke Program Early CT Score (ASPECTS) in 62 acute ischemic stroke patients categorized according to hemorrhagic transformation (HT) and clinical outcome.

	HT (n = 27)	no HT (n = 35)	p value	mRS≤2 (n = 42)	mRS>2 (n = 20)	p value
**Semi-quantitative CTP ASPECTS**						
CBF (median, IQR, mean ± SD, range)	3, 1.5–4.5, 6.1±2.2, 2–9	6, 4.5–8, 3.3±2.4, 0–9	p<0.0001*	6, 3–8, 5.6±2.7, 0–9	4, 2–5, 3.5±2.2, 0–8	p<0.01*
CBV (median, IQR, mean ± SD, range)	7, 6–9, 7.2±1.8, 3–10	9, 9–10, 9.2±1.0, 6–10	p<0.00001^	9, 9–10, 8.8±1.4, 6–10	8, 6–8, 7.3±1.9, 3–10	p<0.01^
MTT (median, IQR, mean ± SD, range)	3, 2–5, 3.6±2.5, 0–9	6, 4–8, 6.0±2.4, 2–9	p<0.001*	6, 3–8, 5.6±2.7, 0–9	4, 2–5, 3.8±2.3, 0–8	p<0.02*
CTP mismatch (median, IQR, mean ± SD, range)	4, 2–5, 3.6±2.0, 0–7	3, 1–5, 3.2±2.3, 0–8	p = 0.488*	1, 1–1, 1.0±0.5, 0–2	4, 2–5, 3.5±2.0, 0–6	p = 0.686*
**Quantitative CTP ASPECTS**						
CBF (median, IQR, mean ± SD, range)	4, 2–6, 3.9±2.6, 0–9	7, 5–8, 6.5±2.2, 2–10	p<0.0001*	7, 4–8, 6.1±2.5, 1–10	4, 2–5, 3.8±2.4, 0–8	p<0.001*
CBV (median, IQR, mean ± SD, range)	9, 7–9.5, 8.1±1.9, 4–10	10, 10–10, 9.6±0.9,6–10	p<0.001^	10, 9–10, 9.3±1.1, 6–10	9, 7–10, 8.1±2.1,4–10	p = 0.021^
MTT (median, IQR, mean ± SD, range)	1, 0–3, 2.0±2.5, 0–9	6, 3–7, 5.1±2.5, 0–9	p<0.0001*	5, 2–7, 4.7±3.0, 0–9	2, 0–2, 1.7±1.7, 0–6	p<0.001*
CTP mismatch (median, IQR, mean ± SD, range)	6, 4–8, 6.1±2.6, 0–10	4, 2,5–7, 4.5±2.6,0–10	p = 0.021*	1, 1–1, 1.0±0.5, 0–2	7, 5–8, 6.4±1.9, 4–10	p<0.02*

CBF = cerebral blood flow; CBV = cerebral blood volume; MTT = mean transit time; SD = Standard deviation; IQR = Interquartile range; mRS ≤ 2 = good outcome; mRS > 2 = poor outcome

statistical analysis = *t-test

^Mann-Whitney.

**Table 3:** Correlation between semi-quantitative and quantitative CT perfusion (CTP) Alberta Stroke Program Early CT Score (ASPECTS) and final infarct volume as measured on NCCT at 7 days after symptom onset in 62 acute ischemic stroke patients considered as a whole (total patients) and grouped according recanalization and reperfusion levels.

	Total patients (n = 62)	Recanalized (n = 45)	not recanalized (n = 17)	Reperfused (n = 25)	not reperfused (n = 37)
**Semi-quantitative CTP ASPECTS**					
CBF	r = -0.582094; p<0.00001	r = -0.500480; p<0.001	r = -0.221318; p = 0.393	r = -0.538078; p<0.01	r = -0.438866; p<0.01
CBV	r = -0.707869; p<0.00001	r = -0.694085; p<0.00001	r = -0.554911; p = 0.021	r = -0.688623; p<0.02	r = -0.682344; p<0.000001
MTT	r = -0.531381; p<0.00001	r = -0.431643; p<0.01	r = -0.138321; p = 0.597	r = -0.483270; p<0.02	r = -0.349885; p = 0.034
CTP mismatch	r = 0.113193; p = 0.381	r = 0.001801; p = 0.991	r = -0.360055; p = 0.156	r = 0.102910; p = 0.624	r = -0.082973; p = 0.625
**Quantitative CTP ASPECTS**					
CBF	r = -0.568907; p<0.00001	r = -0.422621; p<0.01	r = -0.380731; p = 0.132	r = -0.307668; p = 0.135	r = -0.544224; p<0.001
CBV	r = -0.566817; p<0.00001	r = -0.621084; p<0.00001	r = -0.543278; p = 0.024	r = -0.506412; p<0.01	r = -0.602951; p<0.0001
MTT	r = -0.641047; p<0.00001	r = -0.532585; p<0.001	r = -0.419322; p = 0.094	r = -0.479297; p<0.02	r = -0.470496; p<0.01
CTP mismatch	r = 0.313840; p<0.02	r = 0.292660; p = 0.051	r = -0.194174; p = 0.455	r = 0.265259; p = 0.200	r = 0.093855; p = 0.581

CBF = cerebral blood flow; CBV = cerebral blood volume; MTT = mean transit time; statistical analysis = Spearman's rank correlation coefficient.

**Table 4:** Correlation between semi-quantitative and quantitative CT perfusion (CTP) Alberta Stroke Program Early CT Score (ASPECTS) and clinical outcome as measured with modified Rankin scale (mRS) at 3 months in 62 acute ischemic stroke patients considered as a whole (total patients) and grouped according recanalization and reperfusion levels.

	Total patients (n = 62)	Recanalized (n = 45)	not recanalized (n = 17)	Reperfused (n = 25)	not reperfused (n = 37)
**Semi-quantitative CTP ASPECTS**					
CBF	r = -0.329956; p<0.01*	r = -0.263718; p = 0.080*	r^2^ = -0.004; beta = -0.24; p = 0.350^	r = -0.146; p = 0.485*	r = -0.386; p<0.02*
CBV	r = -0.424394; p<0.001*	r = -0.293664; p = 0.050*	r^2^ = -0.233503; beta = -0.53; p = 0.029^	r = -0.037; p = 0.859*	r = -0.572; p<0.01*
MTT	r = -0.301226; p<0.02*	r = -0.204617; p = 0.178*	r^2^ = -0.019; beta = -0.21; p = 0.416^	r = -0.136; p = 0.518*	r = -0.308; p = 0.064*
CTP mismatch	r = 0.065819; p = 0.611*	r = -0.022279; p = 0.885*	r^2^ = -0.061; beta = -0.35; p = 0.175^	r = -0.032; p = 0.879*	r = -0.071; p = 0.0678*
**Quantitative CTP ASPECTS**					
CBF	r = -0.540281; p<0.00001*	r = -0.240467; p = 0.112*	r^2^ = -0.056; beta = -0.34; p = 0.183*	r = -0.101; p = 0.632*	r = -0.445; p<0.01*
CBV	r = -0.609260; p<0.00001*	r = -0.190077; p = 0.211*	r^2^ = -0.214; beta = -0.51; p = 0.035^	r = -0.016; p = 0.941*	r = -0.487; p<0.01*
MTT	r = -0.599370; p<0.00001*	r = -0.381008; p<0.01*	r^2^ = -0.090; beta = -0.38; p = 0.130^	r = -0.297; p = 0.149*	r = -0.407; p<0.02*
CTP mismatch	r = 0.287592; p = 0.023*	r = -0.381008; p<0.01*	r^2^ = -0.047; beta = -0.14; p = 0.604^	r = -0.275; p = 0.183*	r = -0.074; p = 0.665*

CBF = cerebral blood flow; CBV = cerebral blood volume; MTT = mean transit time

statistical analysis = *Spearman's rank correlation coefficient

^Linear regression.

### Semi-quantitative and quantitative ASPECTS optimal values

As reported in Table 5, the optimal cut-off point for predicting favorable outcome was 6 for CBF, 9 for CBV, 6 for MTT and 4 for CTP mismatch in semi-quantitative ASPECTS analysis and 6 for CBF, 9 for CBV, 3 for MTT and 6 for CTP mismatch in quantitative evaluation. CBV ASPECTS at a threshold value of 9 and MTT ASPECTS at a threshold value of 3 were the most accurate parameters to identify good outcome in semi-quantitative and quantitative evaluations, respectively, because they are the CTP ASPECTS scores that gave the best combination of sensitivity and specificity in our patient population.

**Table 5:** Semi-quantitative and quantitative CT perfusion (CTP) Alberta Stroke Program Early CT Score (ASPECTS) optimal values for recognizing AIS patients with good clinical outcome (mRS<2 at 3 months) as calculated using Receiver Operating Characteristic (ROC) curves.

	Cut-off values	Sensitivity	Specificity	AUC
**Semi-quantitative CTP ASPECTS**				
CBF	6	57.1%	80%	0.724
CBV	9	78.6%	75%	0.749
MTT	6	52.4%	75%	0.693
CTP mismatch	4	40.5%	45%	0.455
**Quantitative CTP ASPECTS**				
CBF	6	61.9%	75%	0.754
CBV	9	83.3%	40%	0.682
MTT	3	73.8%	80%	0.793
CTP mismatch	6	38.1%	40%	0.305

mRS = modified Rankin scale; CBF = cerebral blood flow; CBV = cerebral blood volume; MTT = mean transit time; AUC = area under the curve.

## Discussion

In this study, we investigated the prognostic ability and the consistency of semi-quantitative CTP ASPECTS in AIS using a concomitant quantitative method. This is the first time that qualitative and threshold-based quantitative assessments of ASPECTS on CTP maps were compared. In the past, these two techniques were always applied separately to calculate CTP ASPECTS for predicting clinical outcome in AIS patients [13–17]. In addition, our investigation is the first to demonstrate that semi-quantitative CBF, CBV and MTT ASPECTS were higher in AIS patients without HT than in those with HT. On the contrary, there were no differences between HT and no HT in AIS patients for the semi-quantitative ASPECTS mismatch. These findings are in agreement with previous studies documenting that the risk of developing HT is associated not only to low values of CBV [25], but also to reduced levels of CBF and elevated values of MTT [26], and suggest that HT is related to an impairment of the blood-brain barrier promoted by the severity of ischemia, irrespective of the extent of the ischemic penumbra. More importantly, consistent with previously published data [13–17], we proved that semi-quantitative CBF, CBV and MTT ASPECTS were robust predictors of final infarct volume and clinical outcome. In fact, they were greater in patients with mRS≤2 than in those with mRS>2 and their high values were strongly correlated with a small final infarct volume and a favorable clinical outcome. Surprisingly, the semi-quantitative CTP ASPECTS mismatch did not show any association with radiological and clinical outcomes, indicating that the extent of the ischemic penumbra does not seem to be related to the prognosis. These results are discordant with those coming from previous works [15,16], probably due to differences in ASPECTS determination techniques and patient selection. The poor relationship between semi-quantitative CTP ASPECTS mismatch and outcome was confirmed after the classification of patients according to recanalization and reperfusion grades. Moreover, this categorization provided further controversial findings which can explain why the predictive power of semi-quantitative CTP ASPECTS with respect to infarct volume and clinical outcome deteriorated after the stratification of AIS patients according to recanalization and reperfusion statuses. As expected, semi-quantitative CBF, CBV and MTT ASPECTS were inversely correlated with final infarct volume and clinical outcome in recanalized but not in not recanalized patients. Conversely, they showed a negative correlation with final infarct size not only in reperfused but also in not reperfused patients. In addition, an inverse association between semi-quantitative CBF and CBV ASPECTS and mRS≤2 was observed only in not reperfused patients. Therefore, although reperfusion is currently considered a prognostic indicator stronger than recanalization [27], our results suggest that a favorable CTP ASPECTS leads to a good radiological outcome in recanalized patients regardless of reperfusion status, but it is associated with a good clinical outcome only in recanalized and not reperfused patients. This implies that, in some cases, reperfusion may be futile inducing a luxury perfusion, exceeding metabolic demand, in non-viable tissue evolving into infarct [28]. Furthermore, we found that CBV ASPECTS with a cut-off point of 9 was the best semi-quantitative parameter for differentiating between good and poor outcome. This threshold level is in contrast with most prior publications where the optimal value for CBV ASPECTS was >6 [13], ≥7 [15], >7 [16] or ≥8 [14], but it is concordant with a recent paper [17] in which a CBV ASPECTS of 9 was the most accurate for predicting prognosis in a group of AIS patients receiving endovascular treatment. Interestingly, the quantitative analysis performed in our study confirmed that the optimal threshold for CBV ASPECTS was 9, indicating that this threshold could be consistent in identifying favorable prognosis and improving the selection of patients for reperfusion therapies. Of note, this high CBV ASPECTS cut-off value could be affected by the presence of spontaneous recanalization and partial reversibility of CBV lesion. In fact, it has recently demonstrated that CBV ASPECTS can overestimate final infarct size in patients with spontaneous recanalization that occurred in our population given the greater proportion of not treated with respect to not recanalized patients [29]. On the other hand, it has lately been shown that admission CBV volume can be underestimated after early recanalization [29], likely in relation to the appearance of hypervolemia that have the potential to mask infarct tissue [30]. However, our more significant finding is the demonstration that there was a substantial agreement between semi-quantitative and quantitative CTP ASPECTS, which were highly correlated and provided comparable results as indicators of radiological and clinical outcomes. Such data could have important practical implications because they show that visual inspection is a fast, easy and reliable tool to properly define the supposed infarct core (the CBV defect) and the presumed total hypoperfusion (the CBF or MTT defect). In addition, these results further strengthen the hypothesis that the depth of ischemia rather than the extent of the penumbra is the main prognostic determinant in AIS [31]. In fact, while quantitative MTT ASPECTS was the best predictive parameters for clinical outcome, only a slight association was found between quantitative CTP ASPECTS mismatch and radiological and clinical outcome measures. In this regard, the inclusion of tissue not at risk for infarction (benign oligemia) in the quantitative assessment of MTT maps represents a possible explanation for the lower MMT ASPECTS and the higher CTP ASPECTS mismatch we obtained with quantitative analysis compared to visual assessment. MTT thresholds employed in this study to identify total hypoperfusion were not sufficiently restricted to exclude benign oligemia [32]. This study is affected by several limitations. First, the small sample size and the retrospective nature of our investigation could weaken the consistency of our data. Second, our CTP studies were performed with a delay-sensitive deconvolution algorithm and one-phase acquisition protocol which lead to an incorrect estimation of perfusion parameters due to the effects of bolus delay and truncation of time density curves, respectively [30]. However, we chose this approach to be closest to clinical practice since the majority of prior studies were carried-out with a similar protocol [13–17]. Third, the reliability of our quantitative measurements could be questionable since it is well-known that different commercial software programs provide different CTP absolute values [33]. For this reason, we employed thresholds previously validated for one-phase acquisition protocol in our ROI analysis [6,7]. Fourth, the use of the 7 day-NCCT to determine final infarction could lead to an overestimation of final infarct volume due to the development of an ongoing vasogenic edema in early subacute phase. To overcome this possible drawback we had excluded swelling effects from the measurements [22]. Fifth, as recanalization was established using the 24 hour CTA, the exact recanalization time was not known. Finally, the actual impact of ischemic penumbra on outcome remains to be elucidated because we did not use CBF to define infarct core and Tmax to delineate total hypoperfusion, as recently recommended [10,11]. Nevertheless, it is well known that the presence of leukoaraiosis often preclude a correct identification of ischemic core on visual interpretation of CBF maps [10]. In summary, our study showed that visual assessment of CTP ASPECTS represents a good method to recognize the severity of ischemic hypoperfusion and to predict HT and final infarct volume in AIS patients. CBV ASPECTS of 9 was a very stringent threshold value to identify favorable clinical outcome. Conversely, CTP ASPECTS mismatch was a poor prognostic marker suggesting that the extent of ischemic penumbra measured using CBV for delineating infarct core and MTT for defining total hypoperfusion is not related to radiological and clinical outcomes. These findings indicate that size and degree of ischemia as detected by semi-quantitative evaluation of CTP ASPECTS can be used as fast and easily feasible prognostic indicators in AIS patients. Future studies in a larger patient population are warranted to better understand the potential of semi-quantitative CTP ASPECTS in improving the selection of AIS patients for reperfusion therapies.
